# Children infected vs. uninfected with COVID-19: Differences in parent reports of the use of mobile phones to calm children, routines, parent–child relationship, and developmental outcomes

**DOI:** 10.3389/fpubh.2023.1114597

**Published:** 2023-04-13

**Authors:** Eva Yi Hung Lau, Jian-Bin Li, Derwin King Chung Chan

**Affiliations:** Department of Early Childhood Education, The Education University of Hong Kong, Hong Kong SAR, China

**Keywords:** COVID-19, children, infection, routines, parent–child relationships, developmental outcomes, mobile phone use, prosociality

## Abstract

Children were suggested to be at lower risk of developing the severe form of the COVID-19. However, children infected with COVID-19 may be more likely to experience biopsychosocial stressors associated with the pandemic and display poorer developmental outcomes. The current study is among the first to compare children infected and uninfected with COVID-19 on outcomes related to parents’ use of mobile phones to calm children, routines, parent–child relationship, externalizing and internalizing problems, prosocial behavior, gratitude, and happiness. A total of 1,187 parents (88.6% mothers) of children aged 5 to 12 completed an online survey between April 2022 and May 2022 when schools were suspended during the 5^th^ wave of resurgence in Hong Kong. Our findings showed no substantial differences in various psychological, social, emotional, and behavioral outcomes between infected and uninfected children. Our findings can be used to educate parents to reduce their fear and anxieties associated with their children’s COVID-19 infection. Our findings also suggested that support during the pandemic should be provided to children and families regardless of whether children have been infected with COVID-19.

## Introduction

The coronavirus disease 2019 (COVID-19) pandemic, which was caused by acute respiratory syndrome coronavirus 2 (SARS-CoV-2), has infected more than 630 million people worldwide (1). Hong Kong, a city with a population of 7 million, has experienced five waves of the pandemic. The Hong Kong government adopted a dynamic zero-COVID strategy and imposed very strict lockdown measures in each wave of the pandemic, including suspension of face-to-face schooling, mandatory confinement in isolation facilities for those who tested positive for COVID-19, closing of restaurants, bars, and gyms, and occasional ambush lockdowns and compulsory neighborhood-wise testing. Before the last wave of the pandemic in December 2021, Hong Kong only had a total of 12,630 confirmed COVID-19 cases, including 593 children. With the onset of the 5^th^ wave of the pandemic with Omicron variant infections in December 2022, over 2.8 million people, including 225,001 children aged 12 or under, in Hong Kong were infected with COVID-19 (2, 3). While Omicron is the most transmissible variant of SARS-CoV-2, it causes less severe illness than other variants. Children infected with COVID-19 are reportedly asymptomatic or have a milder illness (e.g., fever, cough, fatigue, and diarrhea), lower morbidity, less hospital care, and better prognosis than their adult counterparts (4–7). Nevertheless, young children are more likely than older children to have severe infections and play a major role in community-based viral transmission (e.g., in childcare centers, schools, and homes) (8, 9). In addition to the impact of infection, young children are likely to be vulnerable to the psychosocial stressors associated with pandemic-related environmental restrictions (10). Available evidence has shown that social restrictions implemented to reduce the spread of COVID-19 have led to significant decreases in physical activities, increases in sedentary behaviors, and disrupted sleep patterns in children and adolescents (11–13). The changes are also likely to negatively impact the parent–child relationship at home and children’s social, psychological, and emotional well-being (14).

Existing studies have agreed on children’s vulnerabilities affected by the pandemic (14–16). Regardless of their infection status, children generally experience disrupted daily living routines, prolonged screen product use, increased parent–child conflicts, and heightened socio-emotional and behavioral problems due to school suspension and the lack of social activities. However, it is not clear if the risk of short-and long-term adjustment problems during the pandemic may be higher among those infected with COVID-19. Specifically, children infected with COVID-19 may experience reduced outdoor and social activities as they undergo quarantine. As a result, limited mobility may lead to their lack of interest in other activities, the inability to perform daily routines, and increased externalizing and socio-behavioral problems more than their uninfected counterparts (17). Second, being confined at home due to COVID-19 infection and lacking social support may lead to increased parenting responsibilities and higher parental distress (18–21). When there is a limited choice of activities at home, busy parents may increase their use of mobile phones to calm their infected children to tackle their parenting difficulties, whereas parent–child relationship may also be worsened because of parents’ use of ineffective parenting strategies toward their infected children under infection-related stress. Finally, children and their parents may be concerned about the symptoms of COVID-19 and the long-term health consequences (22). Compared to uninfected children, children with COVID-19 infection may worry more about their health and have a higher level of fear about transmitting the virus to other family members, which could increase their internalizing problems and harm their psycho-emotional well-being.

To our knowledge, only two studies have compared the developmental outcomes of children infected and uninfected with COVID-19 and have produced inconsistent results. Specifically, using a sample of 129 children from 66 families, Costernaro et al. (23) found no differences between infected (*n* = 92) and uninfected (*n* = 37) Italian children’s resilience and behavioral changes, such as externalizing and internalizing problems. It is likely that the nationwide lockdown in Italy during the time that the study was conducted unanimously influenced the outcomes of infected and uninfected children. On the other hand, in their study with 148 Egyptian children, Ahmed et al. (24) found that children with COVID-19 infection (*n* = 36) had a higher percentage of clinical rating on psychological problems, including internalizing and externalizing behaviors, than the uninfected group (*n* = 112). Because of Egypt’s relatively modest lockdown measures (e.g., students were allowed to attend school 2 days a week), infected children may experience more direct negative impacts from the infection above and beyond those impacted by the social restriction measures. Nevertheless, Costernaro et al.’s study was limited by its small sample size, whereas Ahmed et al.’s study did not examine the statistically significant differences between the two groups. As such, a study with a larger sample size with more sophisticated statistical analyzes for a better understanding of the significant differences between infected and uninfected children on psychological, emotional, social, and behavioral outcomes as well as the parent–child relationship is needed. Findings from such a study would help identify the high-risk population and inform prevention and intervention strategies to prevent more severe long-lasting problems in children and families during the pandemic. Due to insufficient evidence from the literature, no specific hypothesis was formulated concerning the outcomes, namely ‘parents’ use of mobile phones to calm children, routine, parent–child conflict, parent–child closeness, prosocial behavior, externalizing and internalizing problems, gratitude, and happiness, between infected and uninfected children.

## Method

### Participants and procedure

The present study is part of a larger longitudinal study on child and family well-being and adjustment during the 5^th^ and last wave of the COVID-19 pandemic in Hong Kong (25). Participants were recruited through kindergartens and primary schools in Hong Kong and *via* a Facebook page managed by the authors’ institution. The findings reported in this paper involved data obtained at the second time point of the study conducted between April 2022 and May 2022. Completed online surveys were received from 1,187 parents of children aged 5 to 12 (Infected = 816; uninfected = 371) *via* Qualtrics. Each respondent was offered HK$50 (~US$6.43) as a token of appreciation for their participation. The participants were primarily mothers (88.6%) aged between 31 and 40 (62.3%) and had an average of 1.46 children aged between 5 and 12 years (*M_age_* = 6.96, SD = 1.95; 50.6% boys). The current sample is mostly middle-class (26). Most of the parents (30.9%) had a monthly household income of HK$20,001-HK$40,000 and had obtained bachelor’s degrees (45.3%). The ethics committee at the authors’ institute reviewed and approved the current study.

### Measures

The measures selected for this study were previously validated with high reliability.

*Routine.* Children’s routine was assessed using the daily living routine subscale (11 items; e.g., *“my child wakes up at about the same time on weekdays*”) from the Child Routine Inventory (27) on a five-point Likert scale (“0 = *almost never*”; “4 = *nearly always*”).

*Parents’ use of mobile phones to calm children.* The use of mobile phones by parents to calm children was assessed using six items (e.g., *“I allow my child to use a mobile phone for peace and quiet in the house”*) developed by Radesky et al. (28). Parents reported on the likelihood of allowing children’s use of phones in various scenarios to calm them down, for peace and quiet, while eating, in public, to keep them occupied while doing chores, or at bedtime. All items were rated on a five-point Likert scale (“1 = *strongly disagree*”; “5 = *strongly agree*”).

*Parent–child closeness and conflict.* Parent–child closeness (7 items; e.g., “*my child openly shares his/her feelings and experiences with me.*”) and conflicts (8 items; e.g., *“my child easily becomes angry at me.*”) were assessed using the subscales from the Child–Parent Relationship Scale (29). All items were rated on a five-point Likert scale (“1 = *completely inapplicable to me*”; “5 = *completely applicable to me*”).

*Prosocial, internalizing, and externalizing behaviors.* Children’s prosocial (5 items; e.g., *“kind to younger children”*), internalizing (10 items; e.g., *“many worries, often seems worried”*), and externalizing (10 items; e.g., *“often lies or cheats”*) behaviors were assessed using the Strengths and Difficulties Questionnaire (30). All items were rated on a three-point Likert scale (“0 = *untrue*”; “2 = *very true*”).

*Gratitude.* Parents’ perception of children’s gratitude was measured using the 6-item Gratitude Questionnaire-6 ((31); e.g., *“my child has so much in life to be thankful for”*) on a seven-point Likert scale (“1 = *strongly disagree*”; “7 = *strongly agree*”).

*Happiness.* Parents’ percepetion of children’s happiness was assessed using a single item (i.e., *“does your child feel happy in general?”*) from the General Happiness Scale (32) on a seven-point Likert scale (“1 = *not at all happy*”; “7 = *extremely happy*”).

## Results

We conducted two analyzes in SPSS to examine whether children not infected with COVID-19 differed in various parent reports of outcomes from those infected with COVID-19. First, we conducted independent t tests to examine the differences in the outcomes between the two groups. Second, we conducted ANCOVAs to further examine the differences between the two groups, controlling for several demographic variables, including child sex, age, report informant, and family socioeconomic status (SES). In both tests, we evaluated the significance not only with *p* values, but also with effect sizes. According to Cohen (33, 34), the Cohen’s *d* values of 0.20, 0.50, and 0.80 represent small, medium, and large effects, respectively, for independent t tests, while the partial eta squared values of.01, 0.06, and.14 represent small, medium, and large effects, respectively, for ANCOVA tests. We considered that the differences would be substantial only when at least a small effect size was achieved.

Descriptive statistics (means and standard deviation), Cronbach’s alpha, and the two comparisons are summarized in Table 1. As summarized in the table, the overall sample showed relatively high levels of parent–child closeness and parent-perceived child happiness, medium levels of routine, parent’s perception of child gratitude, and prosocial behavior, and relatively low levels of parent–child conflict, externalizing and internalizing problems, and parents’ use of mobile phones to calm children. The mean levels of these outcomes between the uninfected and infected groups showed similar patterns.

**Table 1:** Descriptive statistics and comparison of main variables between uninfected and infected children.

Outcomes	Total (*N* = 1,187)		Non-Infected (*N* = 816)		Infected (*N* = 371)		Comparison tests			
	Range	*M* (SD)	*α*	*M* (SD)	*α*	*M* (SD)	*t*(1185) ^b^	Cohen’s d	*F*_(1, 1,181)_ ^c^	*η*^2^_p_
Routine	1–5	3.76 (0.72)	0.87	3.80 (0.71)	0.88	3.68 (0.73)	2.64^**^	0.17	2.86	0.002
Phone use	1–5	1.99 (0.81)	0.86	1.96 (0.82)	0.84	2.06 (0.80)	−2.06^*^	0.13	2.20	0.002
Parent–child closeness	1–5	4.32 (0.58)	0.87	4.33 (0.58)	0.84	4.29 (0.58)	1.22	0.08	0.41	0.000
Parent–child conflict	1–5	2.70 (0.79)	0.85	2.69 (0.80)	0.83	2.71 (0.76)	−0.34	0.02	0.02	0.000
Prosocial behavior	1–3	2.33 (0.41)	0.75	2.34 (0.41)	0.74	2.33 (0.41)	0.37	0.02	0.01	0.000
Externalizing problems	1–3	1.77 (0.37)	0.81	1.76 (0.37)	0.80	1.80 (0.37)	−1.68	0.11	0.94	0.001
Internalizing problems	1–3	1.53 (0.31)	0.68	1.53 (0.31)	0.70	1.52 (0.32)	0.28	0.02	0.60	0.001
Gratitude	1–7	4.66 (0.91)	0.77	4.67 (0.90)	0.80	4.65 (0.97)	0.40	0.03	0.25	0.000
Happiness^a^	1–10	7.97 (1.45)	-	7.98 (1.43)	-	7.96 (1.52)	0.16	0.01	0.29	0.000

a The General Happiness Scale contains only one item, and thus Cronbach’s α was not calculated.

b T refers to independent *t*-tests.

c F refers to ANCOVA analysis, controlling for child sex, age, parental informant, and family SES.

* *p* < 0.05; ** *p* < 0.01.

The results of the independent t-tests suggested that among the nine outcomes, children infected with COVID-19 were reported to show less routine behavior than the uninfected group [*t* (1185) = 2.64, *p* < 0.01, Cohen’s *d* = 0.17]. Moreover, parents of infected children were reported to use mobile phones to calm children more often than those not infected [*t* (1185) = −2.06, *p* < 0.05, Cohen’s *d* = 0.13]. Although these two outcomes showed significant differences based on *p* values, their effect sizes were lower than the small effect (i.e., 0.20), which suggested that these differences, albeit significant, were trivial. In addition, the results of ANCOVAs showed that after controlling for various demographic covariates, no significant differences in any of the examined outcomes were found. This result further suggested that the so-called significant differences found from independent t-tests were not robust if demographic covariates were accounted for. Taken together, these findings indicate that parents of infected and uninfected children with COVID-19 do not show substantial differences in their reports of psychological, social, emotional, and behavioral outcomes in children.

## Discussion

Our study was among the first to compare the significant differences in psychological, social, emotional, and behavioral outcomes and the parent–child relationship between children infected with COVID-19 and those who were uninfected using a large-scale survey. Consistent with Costernaro (2022), we found no differences between the infected and uninfected groups of children in parents’ reports of routines, parents’ use of mobile phones to calm children, parent–child closeness, parent–child conflict, prosocial behaviors, externalizing behaviors, internalizing behaviors, gratitude, and happiness. In the following, we discuss three potential reasons to explain the lack of findings in this study. First, because most children infected with COVID-19 are reportedly asymptomatic or have milder symptoms, the environmental restrictions adopted during the pandemic may have a stronger impact on children’s development than an infection-related impact. To prevent the transmission of COVID-19, most countries have implemented strict social distancing measures. During the data collection for this study, the Hong Kong government imposed school suspension and group gathering limits to reduce the spread of the virus during the largest wave of the COVID-19 pandemic in the city. The suspension of school, limited social life, lack of outdoor activities, and irregular sleeping habits were found to disrupt children’s routines and usual lifestyle, which can lead to poor social and behavioral development in children (17, 19). As a result, the impact of environmental restrictions on children as perceived by parents may be universal and irrespective of whether they were infected with COVID-19.

Second, the stress experienced by the parents in our sample was low, making it less likely for parents to negatively impact the parent–child relationship and child outcomes. Prior studies showed that parents reported positive relationships with family members before, during, or after COVID-19 (35). Consistently, our middle-class sample showed relatively high levels of parent–child closeness and parent-perceived child happiness, medium levels of routine, parent’s perception of child gratitude, and prosocial behavior, and relatively low levels of parent–child conflict, externalizing and internalizing problems, and parents’ use of mobile phones to calm children. It is possible that middle-income families may have more economic resources and a higher-quality of home environment that would reduce the negative infection-related impact on their children’s outcomes. It is also possible that families in Hong Kong may have adapted to the pandemic after several rounds of school suspension since the pandemic outbreak (25). Therefore, being confined at home may not cause significantly more stress for infected children and their families that would affect their subsequent outcomes. Third, the Omicron variant is considered less severe than the previous forms of COVID-19 variants (36). In Hong Kong, only 1.3% of confirmed infections in children under 12 years of age required hospital admission (2, 3). As the symptoms of infected children are mostly mild, children’s worry and fear about their health and the infection may be low. As a result, there may be no significant differences between the reports of parents of infected and uninfected children in internalizing problems and psycho-emotional outcomes such as gratitude and happiness.

### Implications, limitations, and future directions

Other than the impact of COVID-19 infection on children’s health, parents are generally worried about the negative impact of the COVID-19 virus on children’s psychological, emotional, social, and behavioral development (37, 38). Based on our findings, we can reduce parents’ fear and anxieties associated with the impact of COVID-19 infection on their children by offering education to inform them of the lack of differences between the infected and uninfected children on the various outcomes examined. Parents’ reduced concerns and anxieties may further reduce the burden of public health services related to the impact of COVID-19 infection. For instance, previous research has documented the modification in health behavior (e.g., reduction in medical visits and a significant decrease in radiological examinations resulting in negative findings) observed during the pandemic (39). Preventing unnecessary visits to the public health sectors by families with infected children would allow practitioners more time to dedicate to each patient and contribute to a higher quality of health care services. Importantly, our findings also suggested that community family service providers should consider the needs of children and families, regardless of children’s COVID-19 infection status, when developing intervention strategies during the pandemic.

This study has the following limitations to be addressed in future studies. First, we only collected data once. While children and their families may react to the pandemic differently in each wave, it could be helpful if there were longitudinal data to compare the long-term differences between the infected and uninfected groups as well as to account for their prior development. Second, we did not consider other important factors, such as the severity of the symptoms, timing of the COVID-19 infection, perception of the pandemic, parents’ employment status, and parents’ well-being, that may make some infected children more vulnerable to developing more negative outcomes. Third, the participating parents were self-selected to participate in the survey and were considered a middle-class sample. It is possible that the impact of COVID-19 infection may be more severe among children from low-income families because they have fewer resources to buffer against potential hardships (40, 41). As a result, selection bias may have limited the generalizability of our results. Finally, the study was limited by the homogenous use of the parent-report survey and the findings may be biased and only reflect parents’ perception of the outcomes. In particular, because schools were closed when the data was collected, conducting other forms of assessments was not possible. However, parents of infected children may underreport children’s psycho-social adjustment difficulties and regard the outcomes as unrelated to the viral disease to avoid their children being stigmatized for their COVID-19 infection. Future studies should collect information regarding risk factors across multiple time points for a more rigorous comparison of the outcomes between infected and uninfected children over time. Future studies should also achieve a more representative sample and increase the reliability of the data collected by utilizing more diverse recruitment methods (e.g., observation and child tests) and collecting data from multiple informants (e.g., child and teacher reports).

## Data availability statement

The raw data supporting the conclusions of this article will be made available by the authors, without undue reservation.

## Ethics statement

The studies involving human participants were reviewed and approved by The Education University of Hong Kong. Written informed consent to participate in this study was provided by the participants’ legal guardian/next of kin.

## Author contributions

EL acquired funding and resources of the study, conceptualized and wrote the manuscript, and supervised the execution of the study. J-BL performed formal analyzes and wrote the manuscript. DC acquired funding and commented on the draft of the manuscript. All authors contributed to the article and approved the submitted version.

## Funding

This study was partially supported by the Research Cluster Funds of the Department of Early Childhood Education at the Education University of Hong Kong.

## Conflict of interest

The authors declare that the research was conducted in the absence of any commercial or financial relationships that could be construed as a potential conflict of interest.

## Publisher’s note

All claims expressed in this article are solely those of the authors and do not necessarily represent those of their affiliated organizations, or those of the publisher, the editors and the reviewers. Any product that may be evaluated in this article, or claim that may be made by its manufacturer, is not guaranteed or endorsed by the publisher.

## References

[1] WHO. *WHO Coronavirus (COVID-19) Dashboard*. Geneva: World Health Organization. (2022). Available at: https://covid19.who.int/. (Accessed December 2, 2022).

[2] CHP. *Centre for Health Protection of the Department of Health and the Hospital Authority, Statistics on 5th Wave of COVID-19*. (2023). Available at: https://www.covidvaccine.gov.hk/pdf/5th_wave_statistics.pdf. (Accessed March 9, 2023).

[3] CHP. *Centre for Health Protection of the Department of Health and the Hospital Authority, Archives of latest situation of cases of COVID-19*. (2023). Available at: https://www.chp.gov.hk/en/features/102997.html. (Accessed March 9, 2023).

[4] Dong Y Mo X Yabin H Qi X Jiang F Jiang Z. Epidemiology of Covid-19 among children in China. Pediatrics. (2020) 145:e20200702. doi: 10.1542/peds.2020-0702 32179660

[5] Han X Li X Xiao Y Yang R Wang Y Wei X. Distinct characteristics of Covid-19 infection in children. Front Pediatr. (2021) 9:619738. doi: 10.3389/fped.2021.619738, PMID: 33748041 PMC7969512

[6] Shekerdemian LS Mahmood NR Wolfe KK Riggs BJ Ross CE McKiernan CA. Characteristics and outcomes of children with coronavirus disease 2019 (COVID-19) infection admitted to US and Canadian pediatric intensive care units. JAMA Pediatr. (2020) 174:868–73. doi: 10.1001/jamapediatrics.2020.1948, PMID: 32392288 PMC7489842

[7] Wang D Ju XL Xie F Yan L Li FY Huang HH. Clinical analysis of 31 cases of 2019 novel coronavirus infection in children from six provinces (autonomous region) of northern China. Zhonghua Er Ke Za Zhi. (2020) 58:269–74. doi: 10.3760/cma.j.cn112140-20200225-00138 32118389

[8] Cruz AT Zeichner SL. COVID-19 in children: initial characterization of the pediatric disease. Pediatrics. (2020) 145:e20200834. doi: 10.1542/peds.2020-0834 32179659

[9] Tsankov BK Allaire JM Irvine MA Lopez AA Sauve LJ Vallance BA. Severe COVID-19 infection and pediatric comorbidities: a systematic review and meta-analysis. Int J Infect Dis. (2021) 103:246–56. doi: 10.1016/j.ijid.2020.11.163, PMID: 33227520 PMC7679116

[10] Brooks SK Webster RK Smith LE Woodland L Wessely S Greenberg N. The psychological impact of quarantine and how to reduce it: rapid review of the evidence. Lancet. (2020) 395:912–20. doi: 10.1016/s0140-6736(20)30460-8, PMID: 32112714 PMC7158942

[11] Buonsenso D Roland D De Rose C Vásquez-Hoyos P Ramly B Chakakala-Chaziya JN. Schools closures during the COVID-19 pandemic: a catastrophic global situation. Pediatr Infect Dis J. (2021) 40:e146–50. doi: 10.1097/INF.0000000000003052, PMID: 33464019

[12] Chen F Zheng D Liu J Gong Y Guan Z Lou D. Depression and anxiety among adolescents during COVID-19: a cross-sectional study. Brain Behav Immun. (2020) 88:36–8. doi: 10.1016/j.bbi.2020.05.061, PMID: 32464156 PMC7247496

[13] Yeasmin S Banik R Sorif Hossain M Hossain N Mahumud R Salma N. Impact of Covid-19 pandemic on the mental health of children in Bangladesh: a cross-sectional study. Child Youth Serv Rev. (2020) 117:105277. doi: 10.1016/j.childyouth.2020.105277, PMID: 32834275 PMC7387938

[14] Ravens-Sieberer U Kaman A Erhart M Devine J Schlack R Otto C. Impact of the COVID-19 pandemic on quality of life and mental health in children and adolescents in Germany. Eur Child Adolesc Psychiatry. (2022) 31:879–89. doi: 10.1007/s00787-021-01726-5, PMID: 33492480 PMC7829493

[15] Bates LC Zieff G Stanford K Moore JB Kerr ZY Hanson ED. COVID-19 impact on behaviors across the 24-hour day in children and adolescents: physical activity, sedentary behavior, and sleep. Children. (2020) 7:138. doi: 10.3390/children7090138, PMID: 32947805 PMC7552759

[16] Liu Q Zhou Y Xie X Xue Q Zhu K Wan Z. The prevalence of behavioral problems among school-aged children in home quarantine during the COVID-19 pandemic in China. J Affect Disord. (2021) 279:412–6. doi: 10.1016/j.jad.2020.10.008, PMID: 33099056 PMC7543949

[17] Xie X Qi Xue Y Zhou KZ Liu Q Zhang J Song R. Mental health status among children in home confinement during the coronavirus disease 2019 outbreak in Hubei Province, China. JAMA Pediatr. (2020) 174:898–900. doi: 10.1001/jamapediatrics.2020.1619, PMID: 32329784 PMC7182958

[18] Lau EYH Li J-B. Hong Kong 'Children's school readiness in times of Covid-19: the contributions of parent perceived social support, parent competency, and time spent with children. Front Psychol. (2021) 12:779449. doi: 10.3389/fpsyg.2021.779449 34925182 PMC8671738

[19] Lau EYH Lee K. Parents' views on young Children's distance learning and screen time during COVID-19 class suspension in Hong Kong. Early Educ Dev. (2021) 32:863–80. doi: 10.1080/10409289.2020.1843925

[20] Wan AW Hagger MS Zhang C-Q Chung JS Lee K Bautista A. Protecting children from Covid-19: examining U.S. parents motivation and behaviour using an integrated model of self-determination theory and the theory of planned behaviour. Psychol Health. (2022) 2022:1–21. doi: 10.1080/08870446.2022.2111681, PMID: 35975585

[21] Wissemann K Mathes B Meyer A Schmidt NB. Covid-related fear maintains controlling parenting behaviors during the pandemic. Cogn Behav Ther. (2021) 50:305–19. doi: 10.1080/16506073.2021.1878274, PMID: 33787461

[22] Xiong J Lipsitz O Nasri F Lui LMW Gill H Phan L. Impact of Covid-19 pandemic on mental health in the general population: a systematic review. J Affect Disord. (2020) 277:55–64. doi: 10.1016/j.jad.2020.08.001, PMID: 32799105 PMC7413844

[23] Costenaro P Di Chiara C Boscolo V Barbieri A Tomasello A Cantarutti A. Perceived psychological impact on children and parents of experiencing COVID-19 infection in one or more family members. Children. (2022) 9:1370. doi: 10.3390/children9091370, PMID: 36138679 PMC9498072

[24] Ahmed GK Elbeh K Gomaa HM Soliman S. Does Covid-19 infection have an impact on Children's psychological problems? Middle East Curr Psychiatry. (2021) 28:1–9. doi: 10.1186/s43045-021-00155-z

[25] Lau EYH Li J-B Chan DKC. Intention to vaccinate young children against COVID-19: a large-scale survey of Hong Kong parents. Hum Vaccin Immunother. (2022) 18:1–5. doi: 10.1080/21645515.2022.2065838, PMID: 35452345 PMC9302498

[26] Census and Statistics Department. *The Government of the Hong Kong Special Administrative Region, Households*. (2022). Available at: https://www.censtatd.gov.hk/en/scode500.html. (Accessed March 9, 2022).

[27] Sytsma SE Kelley ML Wymer JH. Development and initial validation of the child routines inventory. J Psychopathol Behav Assess. (2001) 23:241–51. doi: 10.1023/a:1012727419873 PMC9734787 36531436

[28] Radesky JS Peacock-Chambers E Zuckerman B Silverstein M. Use of Mobile technology to calm upset children. JAMA Pediatr. (2016) 170:397–9. doi: 10.1001/jamapediatrics.2015.4260, PMID: 26928293

[29] Driscoll K Pianta RC. Mothers' and Fathers' perceptions of conflict and closeness in parent-child relationships during early childhood. J Early Child Infant Psychol. (2011) 7:1–24.

[30] Goodman R. The strengths and difficulties questionnaire: a research note. J Child Psychol Psychiatry. (1997) 38:581–6. doi: 10.1111/j.1469-7610.1997.tb01545.x 9255702

[31] McCullough ME Emmons RA Tsang J-A. The grateful disposition: a conceptual and empirical topography. J Pers Soc Psychol. (2002) 82:112–27. doi: 10.1037/0022-3514.82.1.112, PMID: 11811629

[32] Abdel-Khalek AM. Measuring happiness with a single-item scale. Soc Behav Personal Int J. (2006) 34:139–50. doi: 10.2224/sbp.2006.34.2.139

[33] Cohen J. Statistical Power Analysis for the Behavioral Sciences. Hillsdale, NJ: L. Erlbaum Associates (1988).

[34] Cohen J. Statistical power analysis. Curr Dir Psychol Sci. (1992) 1:98–101. doi: 10.1111/1467-8721.ep10768783

[35] Costenaro P Di Chiara C Boscolo V Barbieri A Tomasello A Cantarutti A. Perceived psychological impact on children and parents of experiencing COVID-19 infection in one or more family members. Children. (2022) 9:1370. doi: 10.3390/children9091370, PMID: 36138679 PMC9498072

[36] Butt AA Dargham SR Loka S Shaik RM Chemaitelly H Tang P. Coronavirus disease 2019 disease severity in children infected with the omicron variant. Clin Infect Dis. (2022) 75:e361–7. doi: 10.1093/cid/ciac275, PMID: 35404391 PMC9047187

[37] De Araújo LA Veloso CF De Campos Souza M De Azevedo JMC Tarro G. The potential impact of the COVID-19 pandemic on child growth and development: a systematic review. J Pediatr. (2021) 97:369–77. doi: 10.1016/j.jped.2020.08.008, PMID: 32980318 PMC7510529

[38] Sinha IP Harwood R Semple MG Hawcutt DB Thursfield R Narayan O. Covid-19 infection in children. Lancet Respir Med. (2020) 8:446–7. doi: 10.1016/s2213-2600(20)30152-1, PMID: 32224304 PMC7154504

[39] Bottari G Stellacci G Ferorelli D Dell’Erba A Aricò M Benevento M. Imaging appropriateness in pediatric radiology during COVID-19 pandemic: a retrospective comparison with no COVID-19 period. Children. (2021) 8:463. doi: 10.3390/children8060463, PMID: 34205841 PMC8227712

[40] George G Dilworth-Bart J Herringa R. Potential socioeconomic effects of the COVID-19 pandemic on neural development, mental health, and K-12 educational achievement. Policy Insights Behav Brain Sci. (2021) 8:111–8. doi: 10.1177/23727322211032248, PMID: 36381537 PMC9648494

[41] Perrigo JL Samek A Hurlburt M. Minority and low-SES families’ experiences during the early phases of the COVID-19 pandemic crisis: a qualitative study. Child Youth Serv Rev. (2022) 140:106594. doi: 10.1016/j.childyouth.2022.106594, PMID: 35845846 PMC9272677

